# Texture analysis in gel electrophoresis images using an integrative kernel-based approach

**DOI:** 10.1038/srep19256

**Published:** 2016-01-13

**Authors:** Carlos Fernandez-Lozano, Jose A. Seoane, Marcos Gestal, Tom R. Gaunt, Julian Dorado, Alejandro Pazos, Colin Campbell

**Affiliations:** 1 Information and Communication Technologies Department, Faculty of Computer Science, University of A Coruna, A Coruna, 15071, Spain; 2 Bristol Genetic Epidemiology Laboratories, School of Social and Community Medicine, University of Bristol, Bristol BS82BN, UK; 3 Stanford Cancer Institute, Stanford School of Medicine, Stanford University, Stanford, 94305, USA; 4 MRC Integrative Epidemiology Unit, School of Social and Community Medicine, University of Bristol, Bristol BS82BN, UK; 5 Instituto de Investigacion Biomedica de A Coruña (INIBIC), Complexo Hospitalario Universitario de A Coruña (CHUAC), A Coruña, 15006, Spain; 6 Intelligent Systems Laboratory, University of Bristol, Bristol BS81UB, UK

## Abstract

Texture information could be used in proteomics to improve the quality of the image analysis of proteins separated on a gel. In order to evaluate the best technique to identify relevant textures, we use several different kernel-based machine learning techniques to classify proteins in 2-DE images into spot and noise. We evaluate the classification accuracy of each of these techniques with proteins extracted from ten 2-DE images of different types of tissues and different experimental conditions. We found that the best classification model was FSMKL, a data integration method using multiple kernel learning, which achieved AUROC values above 95% while using a reduced number of features. This technique allows us to increment the interpretability of the complex combinations of textures and to weight the importance of each particular feature in the final model. In particular the *Inverse Difference Moment* exhibited the highest discriminating power. A higher value can be associated with an homogeneous structure as this feature describes the homogeneity; the larger the value, the more symmetric. The final model is performed by the combination of different groups of textural features. Here we demonstrated the feasibility of combining different groups of textures in 2-DE image analysis for spot detection.

Two-dimensional gel electrophoresis (2-DE) is the method of choice for analysing protein expression in proteomics due to its often-underestimated advantages: robustness, resolution and ability to separate entire proteins at high resolution^1^.

A large number of proteins are separated in the same process according to their electrical charge and molecular mass in order to identify and characterize them. Thus, an array of dark spots on a gel (i.e. polyacrylamide, agarose) is generated (Fig. 1). The analysis of this gel is a non-trivial, tedious and time consuming task, resulting in a bottleneck in proteomics due to differences in protein expression and experimental conditions as well as appearance changes between proteins^2^. Advanced image analysis techniques could improve the performance and quality of this analysis, and one of the key stages of the analysis is the detection of protein spots and differentiation between noise and real protein (Fig. 1). Image classification usually involves computation of features (image attributes) and thus, every image is characterized by attribute vectors with thousands of dimensions. One of these image analysis techniques is texture analysis.

This technique has been widely used in many applications such as remote sensing or document segmentation, for example, but is of special relevance in biomedical imaging for characterization and quantification of different regions of interest (i.e control vs. lesions). There are multiple definition for texture^3^, mainly due to the fact that it is used in a wide array of different applications. In this work textures are defined as the spatial distribution of the grey levels within an image and as a surface’s property can be regarded as the almost regular spatial organization of patterns. Texture is always present even if it is a feature with a low discrimination power.

Thus, image texture is also difficult to analyse. As with many other real-world problems, texture image analysis produces high-dimensional vectors (vectors made up of a large number of features) and it is of relevance to study the importance of each of these features.

Analyzing this collection of high-dimensional data is a challenge. There is also an obvious cost in computational time and memory consumption related with algorithms for analysing high-dimensional data. Furthermore, overfitting may also appear when the number of features exceeds the number of samples^4^ leading to a poor predictive performance. This motivates the development of feature selection techniques to reduce dimensionality. These techniques are aimed at finding the subset of variables that describe in the best possible way the useful information contained in the data, allowing improved performance.

The aim of this work is to find complex combinations of textures that allow the use of the intrinsic information contained within the textures for classification purposes in the best possible way, as well as identifying the more relevant textures for protein classification in 2-DE electrophoresis images. Given there are several different approaches for feature Selection in Machine Learning (ML), in previous work^5^ we chose to evaluate three different machine-learning feature selection approaches: subgroup-based Multiple Kernel Learning, Recursive Feature Elimination with different classifiers (Naïve Bayes, Support Vector Machines, Bagged Trees, Random Forest and Linear Discriminant Analysis) and a Genetic Algorithm based approach with a Support Vector Machines as decision function. Our study reflects that kernel-based approaches improve the interpretation of the results and further investigation should be done in order to find the best combination of variables from different groups of textures in order to measure the particular importance of each one of them to the final solution. Our study should evaluate the power of complex combinations of textures for classification purposes, therefore in this work we will focus in kernel-based methods.

Kernel-based techniques are widely used in bioinformatics and recognized as one of the state-of-the-art classifiers for supervised learning problems due to their ability to encode many different types of data^6^,^7^,^8^,^9^, high test accuracy and ability to deal with high-dimensional datasets^10^. Different types of data can be encoded into kernels, quantifying the similarities of data objects^11^. In particular, for feature selection, these techniques have been widely applied in different areas such as ranking genes functional in cancer^12^, predicting disease progression in breast cancer^13^, microarray data classification^14^ or autism detection^15^. With extensive applications to biomedical appliactions there are a number of reviews^8^,^10^,^16^. One necessary task in any investigation is a experimental evaluation of the performance of a proposed method against comparative state-of-the-art alternatives. The “no free lunch” theorem states that it is impossible to find one algorithm that is the best for solving every problem^17^. Thus, to validate our proposal, an experimental design has been concluded, consisting of four different phases: 1) Data extraction; 2) Data pre-processing; 3) Learning and 4) Selection of the best model to ensure the reproducibility of results.

In this work, a new approach for texture analysis in biomedical imaging is performed by means of integrating different types of features obtained from image textures. For this procedure, kernel-based techniques were used with different types of texture data for the selection of the most representative variables in order to improve the results achieved in classification and the interpretability of complex combinations of textures.

Furthermore, our novel approach allows scientists to investigate whether texture information within the 2-DE electrophoresis images, can be used in the proteomic field to better automatically distinguish spots from noise in the image analysis pipeline.

## Results

### Summary

We assembled six heterogeneous groups of textural features in order to generate the dataset from ten 2-DE images. Thus, we built a training set with 1000 samples and 274 textural variables. Eight different classification models have been used in order to generate the reference/baseline models. The values for each technique were expressed as the (mean ± standard deviation) of the Area Under the ROC curve (AUROC) measure as we performed 10 experiments for each model following a cross-validation scheme.

After the comparison study, we concluded that the integrative kernel-based approach called Feature Selection Multiple Kernel Learning^13^ (FSMKL) for texture analysis is the best integrative technique for finding the best complex combination of textures required to solve the problem. This technique allows us to weight the importance of each particular textural feature in the final model.

### Classification methods comparison

We determined the error plot in (Fig. 2) with four different performance measures (AUROC, Precision, Recall and F-measure. Supplementary Tables 1–4) and the mean ROC curves in (Fig. 3a) with the aforementioned classification models. We started performing experiments with a well-known Naïve Bayes (NB) method, achieving 89.84 ± 0.11%, SVM 89.85 ± 0.11% and SVM applied in the 2D reduced space obtained by SVD-based MCE computed using the correlation norm (ncMCE_corr-SVM) 91.47 ± 0.04%.

We continued performing feature selection (FS) with multiple kernel learning (MKL) (using only the most discriminant group of textures) we could not improve our results (achieving 89.25 ± 0.07%) but this technique seemed more stable, using only fourteen features (wavelet textural features). The same occured if we considered PSO-SVM (achieving 89.13 ± 2.15%), the algorithm cannot escape from local minima in most of the ten experiments performed. Finally we observed improved performance with three other FS approaches, with 94.42 ± 0.48% with a GA-SVM, 95.50 ± 0.16% with FSMKL and 95.74 ± 0.40% with SVM-RFE (an SVM based on recursive feature elimination). The number of features selected for each technique is represented in (Fig. 3b).

SVM-RFE and ncMCE_corr-SVM show high precision at the expense of lower recall than the rest of the models, which means that both models penalize type II errors. This trade-off between precision and recall could be refined choosing different thresholds, and also can be controlled via the use of asymmetric soft margin parameters^18^. These two parameters can be adjusted via validation data and would directly affect the recall, precision and F-measures. Thus, for our stated results, ncMCE_corr-SVM provides a precision and F-measure, which is second best overall, but with worst recall performance (Fig. 2 and Supplementary Tables 2–4). Via use of L1 or L2-norm asymmetric soft margins^19^, we can trade a loss of precision, for a gain in recall, if we wish, depending on our preferred data analysis outcome. The advantage of the AUROC as model selection score is that is independent of the threshold for binarization and includes both type I and type II errors.

### Best model determination

We performed a normality analysis using the Shapiro-Wilk test^20^ with the null hypothesis that the data follow a normal distribution. The null hypothesis was rejected with values *W* = 0.8789 and p-value < 1.74 × 10^−06^, therefore it could be considered that our results did not follow a normal distribution (Fig. 4a). We performed a Bartlett test^21^ with the null hypothesis that our results were heteroscedastic. The null hypothesis was rejected with a value for Barlett’s K squared measure of 150.110 with 7 degrees of freedom and p-value < 2.2 × 10^−16^. In this case, two of the three conditions required for a parametric test does not hold and thus, consistent with both tests, we performed a non-parametric Friedman test with the Iman-Davenport extension assuming the null hypothesis that all models have the same performance. The average rankings of the techniques compared are shown in Table 1 with Iman and Davenport statistic (distributed according to the F-distribution with 7 and 63 degrees of freedom: 78.09 and p-value < 1.30 × 10^−28^). Hence, at this point the null hypothesis is rejected for GA-SVM, SVM, NB, ncMCE_corr-SVM PSO-SVM and MKL with a high level of significance, showing that the winning models are SVM-RFE and FSMKL.

After the test for choose the significantly better models, a Finner^22^ post hoc procedure must be used in order to correct and adjust the *p-values*. Results are shown in (Fig. 4b). Finner’s procedure rejects hypothesis with a value ≤0.043, which means that MKL, PSO-SVM, SVM, NB, ncMCE_corr-SVM and GA-SVM are statistically significantly worse than the winning model. We present for each technique in the comparison in Table 2 the *p-value*, the adjusted *p-value* with Finner procedure and the final value achieved with Finner procedure. We failed to reject the null hypothesis with SVM-RFE and FSMKL. Thus, we performed a non-parametric Friedman test with Iman and Davenport statistic with FSMKL as the control model against NB, MKL, ncMCE_corr-SVM, PSO-SVM, GA-SVM and SVM and we reject the null hypothesis (distributed according to F-distribution with 6 and 54 degrees of freedom: 55.28 and p-value < 2.67 × 10^−21^). Finner’s procedure rejects hypothesis with values value ≤0.050 as shown in (Fig. 4c). For the first image in the dataset we presented in the Supplementary Figures 1–7 the spots wrongly selected during the ten experiments by the best model.

We can conclude that both models behave similarly for this problem, being significantly better than the others, and thus there are no significant differences between FSMKL and SVM-RFE. Those techniques are not differentiable according to their AUROC values. In such case, we perform a pairwise Wilcoxon^23^ test between them according to the final number of features, with the null hypothesis that both models have the same performance and we reject the null hypothesis with a p-value < 7.74 × 10^−06^. As shown in boxplot (Fig. 5), MKL and FSMKL have a lower number of features and are very stable in selecting the features whilst SVM-RFE has outliers, plotted as individual points, and GA-SVM and PSO-SVM selected a higher number of features and have long whiskers indicating variability outside the upper and lower quartiles. Considering this, we finally selected the FSMKL as model of reference.

### Determinant textures

Following an integrative kernel-based approach with FSMKL, we are able to weight the importance of each kernel in the final solution, measuring the relative importance of different groups of textural features in the final decision function. Thus FSMKL can indicate that the calculation of certain types of textural data may not be necessary. We used a large number of kernels, with a variable number of features per kernel. Thus the algorithm finds which kernels, and hence which features per kernel are the most relevant for solving this classification problem^13^.

We observed (Fig. 6) that the final solution is composed of 20 of the 546 kernels initially generated. With the first two kernels we explain 57% of the importance of the final solution. Both kernels only have two features S(0,5)InvDfMom and S(4,0)InvDfMom. With the inclusion of the third kernel in importance, we are able to explain 64% of the importance and we only add one new feature S(3,0)InvDfMom. Those three features are the same, but calculated with different values of distance *d* apart along a given direction with angle Θ. FSMKL selects 23 of the initial 274 textural features extracted as shown in Supplementary Table 5. Once FSMKL finds which kernels (and their particular importance in the final decision function), and which features per kernel, we are able to study the influence of each feature in the final solution as shown in (Fig. 7a), the interactive version is available at http://sabia.tic.udc.es/resources/srep/. The most important feature, S(4,0)InvDfMom, appears in eight kernels with a combined importance weighting of 6.213 (Fig. 7b).

We compare the results of the four FS approaches with a Venn diagram (Fig. 8). This figure shows the overlap among the selected features. The top two features selected by FSMKL (please refer to interactive figure) are only present in the features selected by the SVM-RFE approach, both are statistically significantly better than the others. FSMKL has nine features that no other method uses and from the top ten of features from this method, there are six features that no other method use. Bio-inspired meta-heuristics (GA-SVM and PSO-SVM) are following the same paths in the feature space during its search process as they are sharing 26 features (close to 50% of the features selected by GA).

Furthermore, the most determinant textures are those derived from the Grey Level Coocurrence Matrix (GLCM), absolute gradient and autoregressive model. Second-order statistical features are computed from the intensity of pixels but taking into account spatial relationships of the two pixels in a pair. Extracting information from the GLCM provides information about the intrinsic structure of the texture^24^. The gradient of an image measures the spatial variations of grey levels across the image. Based on a first-order autoregressive model of the image, the model assumes that pixel intensity, in reference to the mean value of image intensity, may be predicted as a weighted sum of four neighboring pixel (left, top, top-left and top-right) intensities. The highest influence is achieved by the Inverse Difference Moment textural feature (IDM) which is a measure of local homogeneity.

## Discussion

Discriminating proteins in 2-DE images is a difficult and challenging task. Our results showed that the combination of textural features using a data fusion approach, taking features from 4 different groups, provides effective classification with very high accuracy. One GLCM feature, IDM (with eight different values of distance *d* and angle *σ*) exhibited high discriminating power. For IDM, a higher value can be associated with a homogeneous or inhomogeneous structure as this feature describes the homogeneity of the ROI texture. It becomes reduced in significance if local textures have maximal change: the larger the IDM, the more symmetric^25^. All the textural features from Autoregressive Model and Absolute Gradient are necessary together with five to nine Histogram features and the previously mentioned GLCM. Most previously published papers consider using only one group of textural features for feature selection strategy^26^.

In biological research, GLCM parameters are generally seen as indicators of structural complexity, heterogeneity and homogeneity^27^. This study demonstrated the feasibility of using texture analysis to characterize the spatial distribution of proteins in 2-DE images in spots and noise.

IDM showed significant distinction between spots, demonstrating that these parameters may be able to differentiate them. The results of this study are consistent with those of previous studies in biomedical image texture analysis, which have reported the importance of second order textural features, in particular IDM, between normal and post-radiation therapy parotid glands in order to assess radiation-induced parotid injury^28^, in the quantification of morphological changes in tissue collagen fibril organization cause by pathological conditions^29^, exploring the cardioprotective effects of schisantherin A in myocardial ischemia-reperfusion (I/R) injury^30^, in the quantification of the inherent heterogeneity of the relationship between biofilms images and the underling processes, such as mass-transport dynamics or substrate concentrations^31^, in the quantification of chromatin structure in kidney in order to demonstrate a loss of complexity on kidney macula densa cells^32^, in the characterization of the zonal dependence of biomechanical changes cause by compressive injury of immature articular cartilage^33^, for plant cortical microtube quantitative analysis^34^, in the quantification of lesion heterogeneity with respect to model-based enhancement kinetics parameters, directly related to tumour physiology^35^ or in the analysis of multiple sclerosis^35^.

High values of GLCM IDM indicate that textural values have minimal changes and are more homogeneous, these values are achieved in the noise. The IDM decreased in the spots (proteins in 2DE-images) indicating that are more inhomogeneous. The smaller the value of IDM, the more difficult the description of the texture because the texture is disorganized. Conversely, the higher the value, the easier the description of texture, because the texture is regular^34^.

For example, the autoreggresive model, absolute gradient and histogram-based textural parameters were also used previously in the literature for non-Hodgkin lymphoma response evaluation with MRI images during treatment with response controlled by quantitative volume analysis^36^, in combination with other texture parameters such as first-, second- and high-order or wavelet for differentiation of adenocarcinoma and gastrointestinal stromal tumors, and between different grades of adenocarcinoma^37^, on either T1- or T2-weighted images for the classification of focal liver lesions on MRI images^38^ or for the classification of multiple sclerosis lesions^39^,^40^, as a potential predictive tool for response evaluation in CT images after the neoadjuvant treatment in patients with lung adenocarcinoma^41^ or aeophageal cancer^42^ and were also used in the quantification of liver fibrosis^43^.

Our results showed that FSMKL-based classification provided an AUROC score at 95.50%. This approach was stastistically significantly different from reference models. In this study, the use of kernel-based techniques was considered for the analysis of high-dimensional input spaces for scenarios such as texture analysis in biomedical imaging. Throughout different kernel-based techniques have been assessed to solve the classification task, both directly and combined with bio-inspired optimization techniques such as genetic algorithms and particle swarm optimization. Such techniques have been proven to solve these issues and, in addition, their use in combination with variable selection techniques renders them very powerful tools. In particular, in this study, kernel-based approaches have been evaluated to select variables via FSMKL (a filter approach), GA and PSO with SVM (wrapper approaches) and SVM-RFE (an embedded approach). The results obtained by FSMKL approach have proven to be significantly better than the others, taking into account the comparison made via different statistical tests. Moreover, as expected, the time required to conduct an experiment with this approach has proven to be the shortest. Furthermore, the winner model (FSMKL) shows that the combination of a kernel integration strategy (MKL) with the feature selection strategy improve the classification results in this texture analysis problem, by combining similar texture features in kernels and selecting the most important ones. This strategy improves also the interpretability of the results, showing the most informative subgroup of textures.

The proposed technique shows how texture analysis can be performed on 2-DE images to classify regions of interest corresponding to spots and noise. This is a very difficult task because of the high inter-and intra-variability (Supplementary Table 6) among different clinicians as they have to manually mark the areas to be studied. With this type of data it can be concluded that, from the entire space of input variables, the texture variable which most strongly enables the distinction between spots and noise, is the *inverse difference moment*, which is a measure of the homogeneity of the image. It is a second-order statistical operator which is calculated from the co-occurrence matrix of grey levels. This study supports the approach that second-order operators in combination with the Autoregressive Model and Absolute Gradient are better for distinguishing between spots and noise in a texture analysis: most of the previously published works considers only one group of textural features^26^.

In summary, we demonstrated the feasibility of combining different groups of textures in 2-DE images images analysis for spot detection. Improved interpretability and the statistically significant difference measured against other state-of-the-art approaches indicate that our approach could be used as part of more complex 2-DE images images analysis pipelines and should be considered for the texture analysis of other types of biomedical images.

## Methods

In this study we compared a set of kernel-based ML methods to see which can obtain better classification performance, and also analyse the nature of the textures selected by the methods.

### The dataset

In order to generate the dataset, ten 1024 × 1024 8-bit 2-DE images^1^ were used, corresponding to an experiment where the effect of a plant extract on the protein expression of IBR3 human dermal fibroblasts was investigated. Spot separation patterns were visualized by silver staining using standard protocols. These images are from the dataset owned by G.-Z. Yang^44^ (Imperial College of Science, Technology and Medicine, London) and have been used in several publications^2^,^45^,^46^.

**Figure 1:**
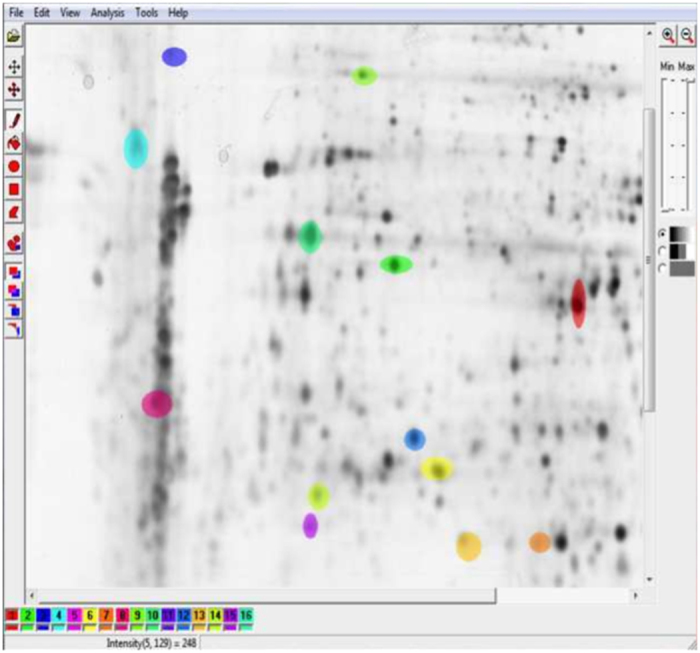
Two-dimensional gel electrophoresis image. Generated using human dermal fibroblasts in order to study the effect of a plant extract on the protein expression of IBR3. 1024 × 1024 8-bit image. 2D protein separation were visualized by silver staining using standard protocols. From the dataset of G.-Z. Yang and co-workers^44^. Spots manually segmented using Mazda software, first step in the image analysis pipeline (dataset generation).

**Figure 2:**
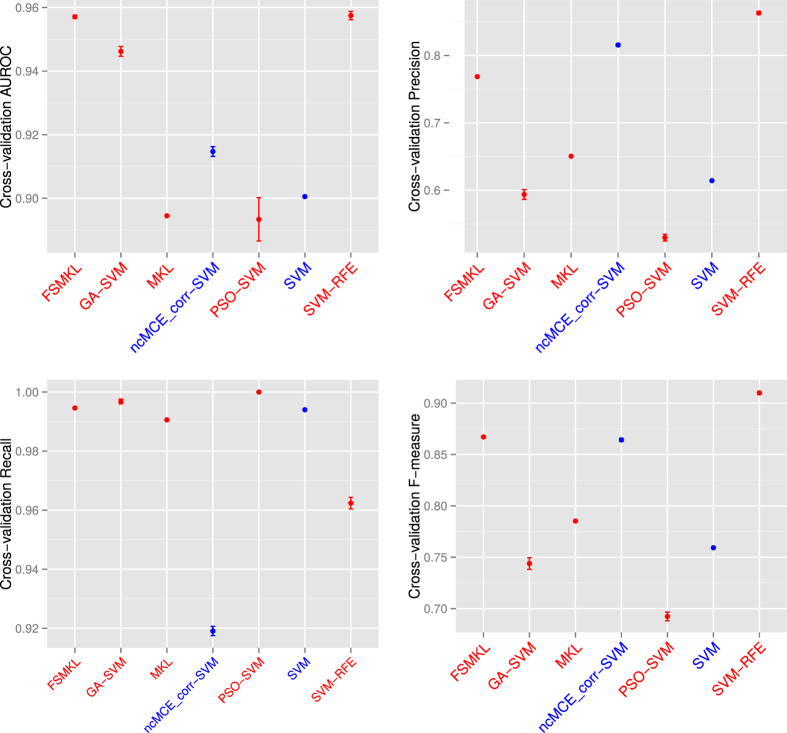
Classification models comparison during 10 experiments. Cross-validation AUROC, Precision, Recall and F-measure values error plot. Machine Learning techniques with Feature Selection are in red, without Feature Selection are in blue.

**Figure 3:**
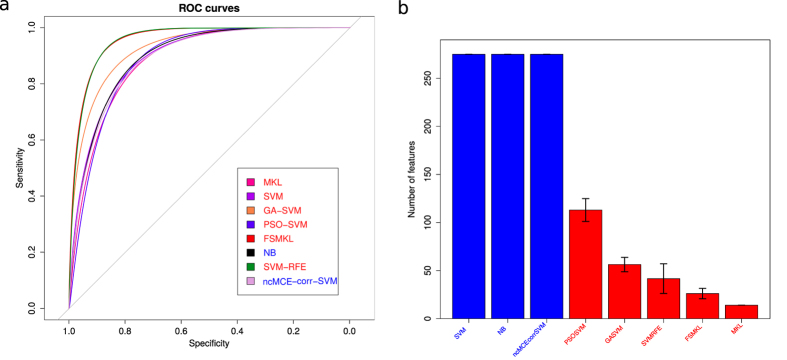
Classification models comparison during 10 experiments. (**a**) ROC curves. (**b**) Final number of features selected for each technique. Machine Learning techniques with Feature Selection are in red, without Feature Selection are in blue.

**Figure 4:**
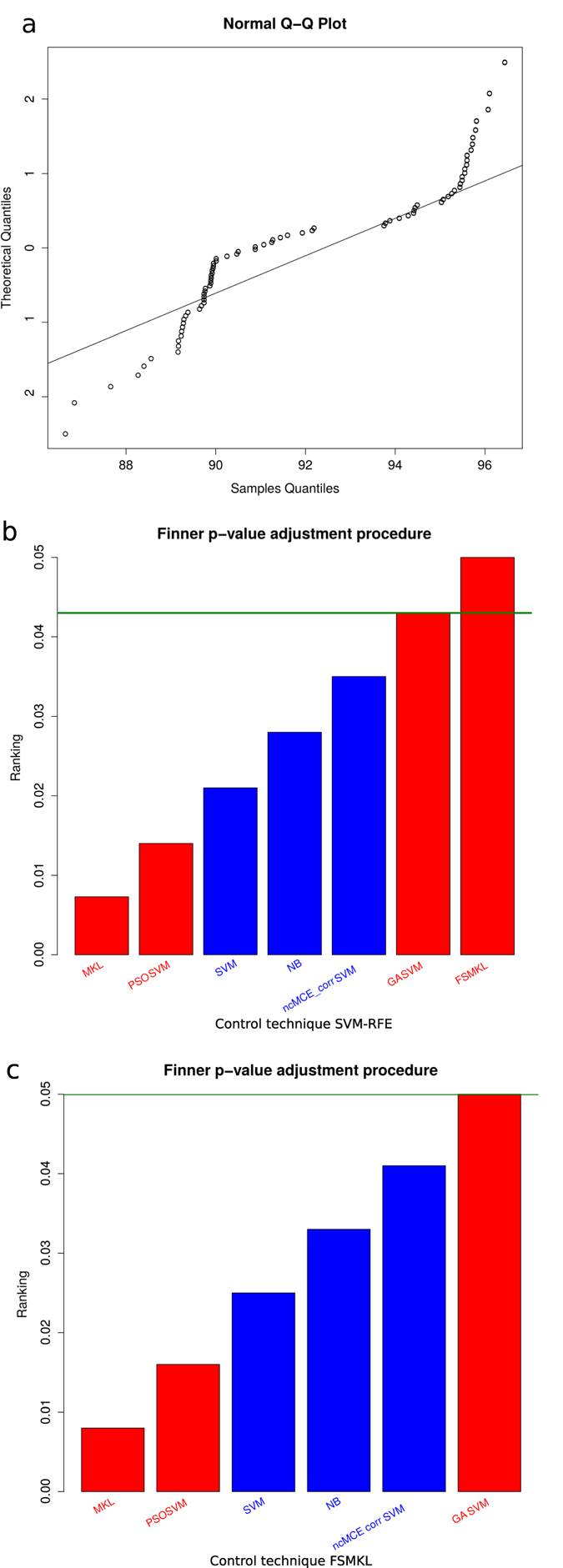
Best model determination. (**a**) Q-Q plot of observed versus expected values. (**b**) Finner p-value adjustment post hoc procedure. Control technique: SVM-RFE. (**c**) Finner p-value adjustment post hoc procedure. Control technique: FSMKL. Machine Learning techniques with Feature Selection are in red, without Feature Selection are in blue.

**Figure 5:**
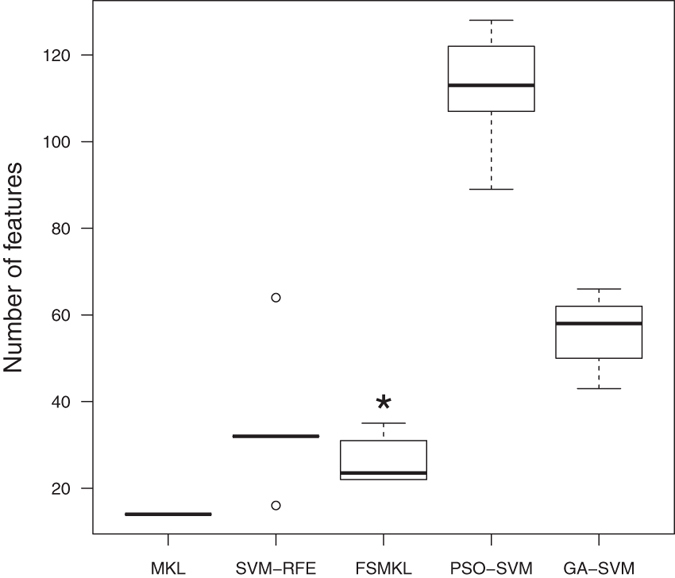
Number of features selected by each technique during the experimentation. This boxplot shows the stability and the number of features by each technique during the cross-validation process of the ten experiments. *Statistically significant difference with a p-value < 7.74 × 10^−06^ according with a pairwise Wilcoxon test: SVM-RFE and FSMKL.

**Figure 6:**
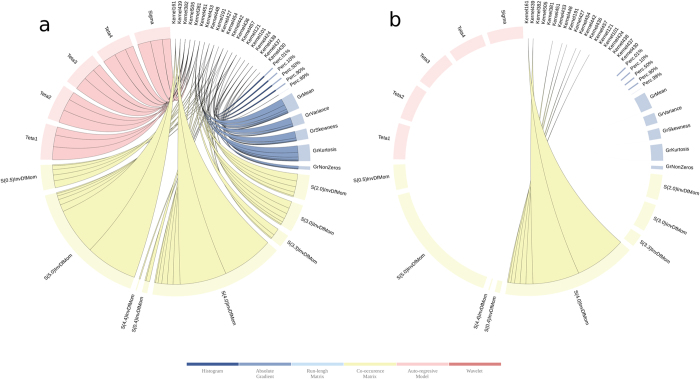
Importance of the kernels in the final solution in a FSMKL approach. This figure shows the importance of each kernel in the final solution. Non-zero values indicate the informative kernels. For each group of textural features we used several different types of kernels, the final decision function were only considering polynomial (with 1 and 2 degrees of freedom) and Gaussian kernels (with low values for the free parameter). The interactive version is available at http://sabia.tic.udc.es/resources/srep/.

**Figure 7:**
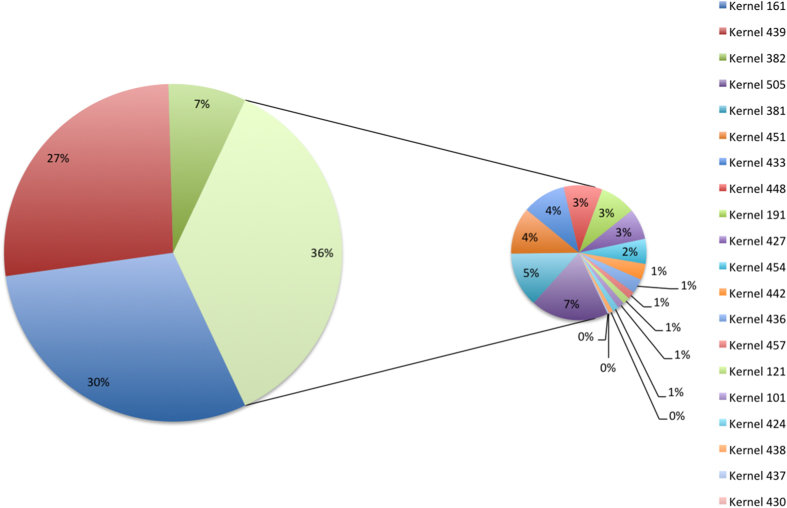
Importance in the final solution in a FSMKL approach. (**a**) This figure shows the importance of each feature in the final solution and the particular kernel in which the feature appears. Inverse difference moment (InvDfMom) is influenced by the homogeneity of the image. (**b**) This figure shows the importance of feature S(4,0)InvDfMom in the final solution and the particular kernels in which the feature appears. This feature appears in eight kernels with a combined weight importance of value 6.213.

**Figure 8:**
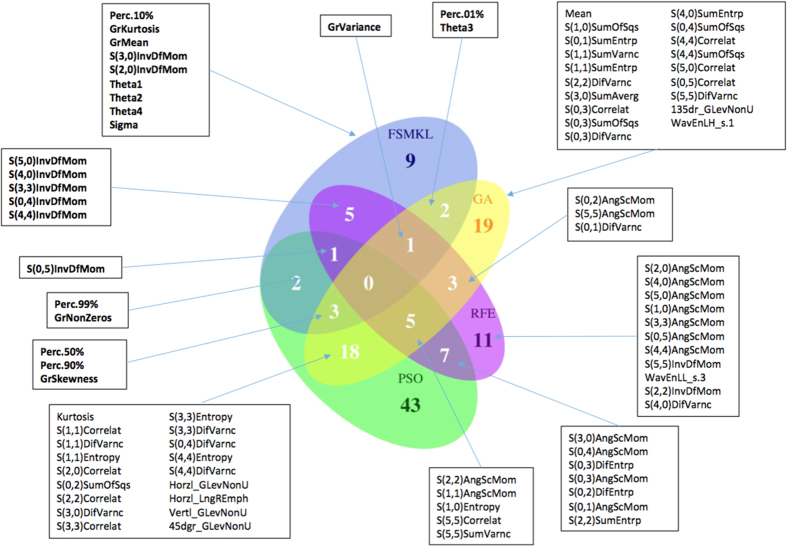
Venn diagram for the results obtained by different Feature Selection methods (from the R library VennDiagram). Comparing FSMKL, SVM-RFE, GA-SVM and PSO-SVM methods by checking the overlaps of their selected textural features (Top features are in bold character). Labels for the Histogram features: Perc. = percentile derived from the image histogram, Mean and Kurtosis. Labels for the absolute gradient features: Gr. = absolute gradient parameters (kurtosis, mean, skewness, variance and and percentage of pixels with nonzero gradient). Labels for the run-length matrix features: GLevNonU = grey-level non-uniformity, LongREmph = long-run emphasis. Calculated for vertical, horizontal, 45-degree and 135-degree directions. Labels for the co-occurrence matrix features: values in parenthesis represent coordinates, containing information about distance and direction between pixels (InvDfMom = inverse difference moment, AngScMom = angular second moment, DifEntrp = difference entropy, SumEntrp = sum entropy, Entropy, Correlat = correlation, SumVarnc = sum variance, DifVarnc = difference variance, SumOfSqs = sum of squares, SumAverg = sum average). Labels for the Autoregressive model features: Theta and Sigma. Labels for the Wavelet features: WavEnLH = energy of wavelet coefficients in subband LH, WavEnLL = energy of wavelet coefficients in subband LL.

For each image out of these ten, two different clinicians agreed (inter- and intra-variability in Supplementary Table 6) on 100 regions of interest (ROI), 50 spots representing proteins and 50 representing noise (noise, background, non-protein regions, cracks) manually segmented that were selected to build a training set with 1000 samples and 274 textural features using Mazda iteratively (Fig. 1). Each ROI were selected taking into consideration that there is an area of influence surrounding it, so it is slightly bigger than the visible black surface of the spot because texture information could exist also in the grey levels closest to white. Thus, one image is studied as shown in Supplementary Figure 1 where all the ROIs computed by Mazda are mixed in the same image.

We preprocess this dataset in order to have a standard normal distribution (a mean of zero and a standard deviation of one). The dataset is available for download at http://dx.doi.org/10.6084/m9.figshare.1368643.

### Texture measures extraction

Texture can be determined in terms of patterns of homogeneity in appearance among spatially close pixels. Textural variables can be calculated by means of three different approaches: statistical, model-based and transform methods^3^,^47^,^48^. Statistical variables include those derived from grey-level co-occurence^49^ and run-length matrix as well as mean, variance, skewness or percentiles derived from the image histogram. Model-based approaches interpret texture variables using different models such as the stochastic-like auto-regressive model. Finally, transform methods decompose an image in terms of frequency or wavelet: Gabor, Fourier or Wavelet transforms^50^.

We considered six groups of textural features: Histogram-based (first-order statistical texture features), Absolute Gradient, Run-length Matrix (high-order statistical texture features), Co-occurrence Matrix (second-order statistical texture features), Autoregressive Model and Wavelet. These features are based on image histogram, co-occurrence matrix (information about the grey level value distribution of pairs of pixels), image gradients (spatial distribution of grey level values), auto-regressive models (description of texture based on statistical correlation between pairs of pixels) and wavelet analysis (information about image frequency at different scales). A more detailed description of those groups is available in the Supplementary Materials document.

We calculated those features using the specialized software *Mazda*^51^. Various approaches have demonstrated the effectiveness of this software, extracting textural features in different types of medical images^51^,^52^. A good review is provided in^53^. Final features used in this work are shown in Supplementary Table 7.

### Machine learning techniques without feature selection

We started our experiments using two well-known machine learning methods for establishing the baseline AUROC performance for comparison. In this case we used NB^54^ and SVM^55^. These techniques are suitable to deal with high-dimensional datasets. We performed our experiments with the Weka^56^ implementation of the Naive Bayes algorithm. This algorithm is usually considered a naive approach because it assumes conditional independence and normal distribution in each class for the attributes. For more information about NB, please refer to^57^. The basic implementation of the SVM is for a binary (two well-separated classes) classification problem separating data via a hyperplane with maximal separation between the closest data points on each side (the support vectors). For more information about SVMs, we refer to^58^,^59^. For SVM and MKL we perform our experiments using MATLAB and SimpleMKL^60^ code. We also included a novel SVM applied in the 2D reduced space obtained by SVD-based MCE computed using the correlation norm^61^,^62^.

### Feature selection

In high-dimensional spaces it is usual to perform a feature selection approach in order to reduce the number of features and to improve the performance of the algorithms. There are mainly three different approaches: Filter (assess the relevance of the features by looking at the intrinsic properties of the data ignoring the model), wrapper (embed the model and the feature subset search) and embedded (the feature subset search is built into the model construction) methods are the three main approaches^63^. In this study we perform experiments with two filter (MKL^64^ filtering groups of features and FSMKL), two wrapper (PSO and GA with an SVM as decision function) and one embedded method (SVM-RFE) methods.

A kernel is a function that maps the input into a higher dimension in order to find a new space where the data are linearly separable. However, kernel functions and its parameters have to be determined. Thus, MKL provides a general framework for learning from multiple groups of data^65^, encoding those groups in different kernels and combining kernels in a final decision function, each one with its particular value of importance^18^ and automatically select these kernels and parameters. For more information about kernel-based learning machines please refer to^6^,^66^.

PSO^67^ and GA^68^ are bio-inspired optimization meta-heuristics for finding the best subset^69^ of input features that best reproduce the original structure of the data. PSO is based on the simulation of the social behavior of bird flocks, during its execution a set of particles moves within the function domain searching for the best fitness value whereas GA are inspired by Darwinian Evolution and an initial population of individuals (possible solutions within the function domain of a fitness function to be optimized) is evolved by means of genetic operators. Based on the latest Standard PSO implementation (SPSO-2011)^70^,^71^ and GAlib^72^, we modify them in order to add a binary representation for each particle/individual as a feature mask for the input feature space. SVM decision function is obtained by LIBSVM^73^.

SVM-RFE^12^ was originally developed for ranking genes in a cancer classification problem according to the hyperplane decision value of a SVM. RFE operates by removing genes (one or more at each iteration) according to the lowest score until the highest performance is achieved. The R Statistical Package^74^ was used and it was necessary to enhance the Caret package^75^ capacities in order to include a new ranking criterion 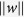 for supporting SVM-RFE as initially proposed by Guyon^12^, we used Kernlab^76^ and pROC^77^ for ROC curves and bar plot figures. Non-parametric tests were performed using code provided by Garcia^17^ *et al.* and are available in the Supplementary Materials of this work.

The FSMKL model presented in^13^ used a sparse MKL minimization algorithm, which allowed selection of a low number of kernels and their ranking by importance for classification. For each of the datasets (in this particular case, for each group of textures), the FSMKL ranks the features of each group of textures in relation to the most statistically aligned with the class, and codes subsets of these ranked features as kernels. In this way, the FSMKL select, not only the most important texture subgroup for classification, but also a subset of the textures which compose that group. In this work, from the six textural groups presented, this method allows to find the most relevant texture features and groups for the given classification problem. Furthermore, FSMKL gives greater interpretability to the complex relationships among different groups of texture than simply performing the better feature selection combination as this technique can measure the final importance of each feature to the final solution.

### Experimental analysis

In order to discover which of the proposed models are statistically significantly better, a set of tests was performed for the analysis of the behaviour of those techniques following the methodology proposed in^17^,^78^,^79^. In order to choose between a parametric or a non-parametric test to compare the models, we used three required conditions for using parametric tests: independence, normality and heteroscedasticity. The use of a parametric test is only appropriate when the results of those techniques fulfilled the three conditions aforementioned^17^.

The independence condition is fulfilled because we perform different runs following a tenfold cross-validation approach for separating the data with a prior random reshuffling of the examples. A cross-validation approach splits the dataset into ten random equal-size subsets, nine of which are chosen ten times to train the model and the remaining set is used to test them (each iteration a random subset is chosen to be the test set).

For the normality condition we used the Shapiro-Wilk test^20^ with the null hypothesis that the data follow a normal distribution and in order to evaluate the heteroscedasticity, we performed a Bartlett test^21^ with the null hypothesis that the results were heteroscedastic. In order to compare the models, a non-parametric Friedman test with the Iman-Davenport extension was employed, where the null hypothesis is that all the models have the same performance. Once the test for check if a model is statistically better than the others, a post-hoc procedure had to be used in order to address the multiple hypothesis testing among the different models. A Finner^22^ post-hoc procedure has to be used for detecting significance of the multiple comparisons^17^,^79^,^80^ and the *p-values* should be corrected and adjusted. We perform our experimental analysis with a level of confidence = 0.05.

At this point, if we failed to reject the null hypothesis for two or more models, we can conclude that those models behave similarly for this problem and that there are no significant differences between them. The test reports in this case that those techniques are not differentiable with the particular performance measure used. In such case it is possible to keep the first one according to the ranking, considering that is the number one in the ranking but it is no significantly better that the others or it is possible to perform a new test taking into consideration other performance measure (number of features, time or simplicity depending on the particularities of the algorithms)^17^,^80^.

## Additional Information

**How to cite this article**: Fernandez-Lozano, C. *et al.* Texture analysis in gel electrophoresis images using an integrative kernel-based approach. *Sci. Rep.* **6**, 19256; doi: 10.1038/srep19256 (2016).

## Supplementary Material

Supplementary Information

## Figures and Tables

**Table 1:** Friedman’s average ranking.

Technique	Ranking
SVM-RFE*	1.29
FSMKL*	1.70
GA-SVM*	2.99
ncMCE_corr-SVM	4.1
SVM	5.89
NB	5.89
PSO-SVM*	6.7
MKL*	7.4

^*^Average rankings of the different techniques used in this study using non-parametric Friedman test with Iman and Davenport extension. The lower the ranking, the better result: SVM-RFE is the control model. Feature Selection approach.

**Table 2:** Adjusting p-values with Finner post hoc procedure.

Finner score for SVM-RFE as winner				Finner score for FSMKL as winner		
Technique	p-value	adjusted p-value	Finner score	p-value	adjusted p-value	Finner score
SVM-RFE*	–	–	–	–	–	–
FSMKL*	0.681	0.681	0.05	–	–	–
GA-SVM*	0.100	0.116	0.043	0.300	0.300	0.050
ncMCE_corr-SVM	3.00 × 10^−3^	4.20 × 10^−3^	0.035	0.029	0.035	0.041
NB	2.60 × 10^−4^	6.08 × 10^−4^	0.028	5.41 × 10^−5^	1.08 × 10^−4^	0.033
SVM	2.60 × 10^−4^	6.08 × 10^−4^	0.021	5.41 × 10^−5^	1.08 × 10^−4^	0.025
PSO-SVM*	5.01 × 10^−6^	1.75 × 10^−5^	0.014	1.14 × 10^−6^	3.43 × 10^−6^	0.016
MKL*	5.14 × 10^−7^	3.60 × 10^−6^	0.0073	2.27 × 10^−8^	1.36 × 10^−7^	0.085

^*^In this table we present the p-value, adjusted p-value and the final value achieved with Finner procedure for the techniques evaluated in this study according with the winner model. Feature Selection approach.
