# Supplementary material for: Texture analysis in gel electrophoresis images using an integrative kernel-based approach
Source: Sci Rep. 2016 Jan 13;6:19256. doi: 10.1038/srep19256 (PMC4713050; doi:10.1038/srep19256)
Supplement: Supplementary Information [file srep19256-s1.pdf]

Supplementary Materials: Texture analysis in gel electrophoresis images using an integrative kernel-based approach

Carlos Fernandez-Lozano[1,*], Jose A. Seoane[2,3], Marcos Gestal[1], Tom R. Gaunt[4], Julian Dorado[1], Alejandro Pazos[1,5], and Colin Campbell[6]

[1]Information and Communication Technologies Department. Faculty of Computer Science, University of A Coruña, A Coruña, 15071, Spain
[2]Bristol Genetic Epidemiology Laboratories. School of Social and Community Medicine, University of Bristol, Bristol BS8 2BN, UK
[3]Stanford Cancer Institute. Stanford School of Medicine. Stanford University. Stanford, 94305, USA
[4]MRC Integrative Epidemiology Unit. School of Social and Community Medicine, University of Bristol, Bristol BS8 2BN, UK
[5]Instituto de Investigacion Biomedica de A Coruña (INIBIC), Complexo Hospitalario Universitario de A Coruña (CHUAC), A Coruña, 15006, Spain
[6]Intelligent Systems Laboratory, University of Bristol, Bristol BS8 1UB, UK
[*]carlos.fernandez@udc.es

ABSTRACT

Texture information could be used in proteomics to improve the quality of the image analysis of proteins separated on a gel. In order to evaluate the best technique to identify relevant textures, we use several different kernel-based machine learning techniques to classify proteins in 2-DE images into spot and noise. We evaluate the classification accuracy of each of these techniques with proteins extracted from ten 2-DE images of different types of tissues and different experimental conditions. We found that the best classification model was FSMKL, a data integration method using multiple kernel learning, which achieved AUROC values above 95% while using a reduced number of features. This technique allows us to increment the interpretability of the complex combinations of textures and to weight the importance of each particular feature in the final model. In particular the *Inverse Difference Moment* exhibited the highest discriminating power. A higher value can be associated with an homogeneous structure as this feature describes the homogeneity; the larger the value, the more symmetric. The final model is performed by the combination of different groups of textural features. Here we demonstrated the feasibility of combining different groups of textures in 2-DE image analysis for spot detection.

MaZda analysis software and texture parameters

MaZda[1] is a computer program for calculation of texture features in digitized images. The program code has been written in C++. This software allows computation of a variety of parameters derived from image histogram, absolute gradient, run-length matrix, co-occurrence matrix, autoregressive model and Haar wavelet groups (Table 7).

The following information is extracted from the Mazda user's manual and author's publication.[1]

First-order histogram

First-order or histogram-based textural features are computed directly from the intensity of pixels and using no information about the spatial relationships between them. The histogram of an image is the count of the number of pixels for a given gray level value.

Textural features are statistical parameters of the histogram distribution: mean brightness, variance, skewness, kurtosis and percentiles. Another statistical method derives features from the gradient magnitude map of the image. Please refer to Mazda user's manual for the particular equations of these features.

Second-order histogram

Computed from the intensity of pixels but taking into account spatial relationships of the two pixels in a pair is defined. For each pair of pixels it is computed across a particular direction and distance among them. Those features are derived from the

co-occurrence matrix: angular second moment, contrast, correlation, sum of squares, and various averages, variances, inverse moments and entropies.[2] Please refer to Mazda user's manual for the particular equations of these features.

The highest influence in this work is achieved by the Inverse Difference Moment textural feature (IDM) which is a measure of local homogeneity with the following equation

$$IDM = \sum_{i=1}^{G-1} \sum_{j=1}^{G-1} \frac{1}{1+(i-j)^2} P(i,j) \tag{1}$$

where G is the number of grey levels used, and from the Gray Level Coocurrence Matrix (GLCM) each element contains the second order statistical probability value from changes between grey levels i and j at a particular distance d and at a particular angle Θ having particular (co-occurring) values i and j. The matrix element $P(i,j \mid \Delta x, \Delta y)$ is the relative frequency with which two pixels, separated by a pixel distance $(\Delta x, \Delta y)$, occur within a given neighborhood and with intensities i and j.

According with this equation, a low IDM value is achieved with inhomogeneous images and a relatively higher value with homogeneous images. Reviewing our dataset, Proteins have low IDM values (inhomogeneous) and Noise have high IDM values (homogeneous).

Run-length matrix

Across a given direction, the run-length matrix measures how many times there are runs of consecutive pixels with the same gray level value. In this software there are four run-length matrices computed, for four directions of pixel runs: horizontal, vertical, at 45° and at 135°. MaZda calculates five different textural features from this matrix: short run emphasis, inverse moment, long run emphasis moment, gray-level non-uniformity, run length non-uniformity and fraction of image in runs.[2] Please refer to Mazda user's manual for the particular equations of these features.

Model-based textural features

Based on a first-order autoregressive model of the image. The model assumes that pixel intensity, in reference to the mean value of image intensity, may be predicted as a weighted sum of four neighboring pixel (left, top, top-left and top-right) intensities. This group is aimed to find relations between neighborhood of pixels (shapes within the image). Please refer to Mazda user's manual for the particular equations of these features.

Absolute gradient

The gradient of an image measures the spatial variations of gray levels across the image. A high gradient value is achieved with an abrupt variation of gray level value (for example from black to white). Those features are derived from the gradient: mean, variance, skewness, kurtosis and percentage of pixels with nonzero gradient. Please refer to Mazda user's manual for the particular equations of these features.

Discrete Haar Wavelet

Wavelets analyzes the frequency of an image in different scales. The wavelet images are scaled up to five times, both in horizontal and vertical direction. It results in image transformation into 20 frequency channels. Please refer to Mazda user's manual for the particular equations of these features.

The dataset

In order to generate the dataset, ten 1024 × 1024 8-bit 2-DE images[3] were used, corresponding to an experiment where the effect of a plant extract on the protein expression of IBR3 human dermal fibroblasts was investigated. Spot separation patterns were visualized by silver staining using standard protocols. These images are from the dataset owned by G.-Z Yang[4] (Imperial College of Science, Technology and Medicine, London) and have been used in several publications.[5–7]

For each image out of these ten 100 regions of interest (ROI), 50 spots representing proteins and 50 representing noise (noise, background, non-protein regions) manually segmented that were selected to build a training set with 1000 samples and 274 textural features. We preprocess this dataset in order to have a standard normal distribution (a mean of zero and a standard deviation of one). The dataset is available for download at http://dx.doi.org/10.6084/m9.figshare.1368643.

We also included in the Supplementary Materials the Mazda (.roi) files and the images in order to reproduce the manual segmentation process and also to point out the particular spots selected for each image. This information is available for download at http://dx.doi.org/10.6084/m9.figshare.1538606.

With Mazda it is only possible to define up to 16 regions of interest for each image, so there exist eight (.roi) files for each image. Please refer to Mazda user's manual for the particular instructions to load an image and (.roi) files.

References

1. Szczypiński, P. M., Strzelecki, M., Materka, A. & Klepaczko, A. Mazda—a software package for image texture analysis. *Computer Methods and Programs in Biomedicine* **94**, 66 – 76 (2009).
2. Haralick, R. Statistical and structural approaches to texture. *Proceedings of the IEEE* **67**, 786–804 (1979).
3. Rabilloud, T., Chevallet, M., Luche, S. & Lelong, C. Two-dimensional gel electrophoresis in proteomics: Past, present and future. *Journal of proteomics* **73**, 2064–77 (2010). URL http://www.sciencedirect.com/science/article/pii/S1874391910001752.
4. Veeser, S., Dunn, M. J. & Yang, G.-Z. Multiresolution image registration for two-dimensional gel electrophoresis. *PROTEOMICS* **1**, 856–870 (2001).
5. Dowsey, A. W. *et al.* Image analysis tools and emerging algorithms for expression proteomics. *Proteomics* **10**, 4226–57 (2010).
6. Rodriguez, A., Fernandez-Lozano, C., Dorado, J. & Rabuñal, J. R. Two-dimensional gel electrophoresis image registration using block-matching techniques and deformation models. *Analytical biochemistry* **454**, 53–9 (2014). URL http://www.sciencedirect.com/science/article/pii/S0003269714000840.
7. Fernandez-Lozano, C., Seoane, J., Gestal, M., Gaunt, T. & Campbell, C. Texture classification using kernel-based techniques. In Rojas, I., Joya, G. & Gabestany, J. (eds.) *Advances in Computational Intelligence*, vol. 7902 of *Lecture Notes in Computer Science*, 427–434 (Springer Berlin Heidelberg, 2013). URL http://dx.doi.org/10.1007/978-3-642-38679-4_42.

Acknowledgements

This work is supported by "Collaborative Project on Medical Informatics (CIMED)" PI13/00280 funded by the Carlos III Health Institute from the Spanish National plan for Scientific and Technical Research and Innovation 2013-2016 and the European Regional Development Funds (FEDER), UK Medical Research Council (G10000427, MC_UU_12013/8) and "Development of new image analysis techniques in 2D Gel for Biomedical research" (ref.10SIN105004PR) funded by Xunta de Galicia. The authors thank the Galicia Supercomputing Centre (CESGA) for the provision of computational support. The authors also thank Dr. G.-Z Yang for providing the dataset and Dr. S. García for supporting in the statistical testing of this paper.

Table 1. Cross-Validation AUROC

Method	AUROC	Standard deviation	Standard error	Confidence interval
FSMKL	0.957089999999999	0.001689172052285341	0.000534163104512271	0.001208360892974959
GA	0.946206799999998	0.004902252742929042	0.001550228433346367	0.003506860354467924
MKL	0.894499999999998	0.000711225546347904	0.000224909265655703	0.000508780106282732
ncMCESVM	0.914715200000000	0.004887895543528357	0.001545688288253630	0.003496589832726246
PSO	0.893379599999999	0.021533306951480279	0.006809429552221455	0.015403999836127537
SVM	0.900538399999999	0.001138610771461838	0.000360060340622086	0.000814513078577812
SVM-RFE	0.957469599999999	0.004076704202607279	0.001289167062701955	0.002916298504934749

In this table we show for each classification methods (10 experiments) the mean, standard deviation, standard error of the mean and the confidence interval multiplier for standard error measures achieved.

Table 2. Cross-Validation Precision

Method	Precision	Standard deviation	Standard error	Confidence interval
FSMKL	0.768632257736836	0.00265309885023290	0.000838983522430991	0.00189791258473693
GA	0.593637760835108	0.02292878550468730	0.007250718617626519	0.01640226505629813
MKL	0.650433312350657	0.00230298042246553	0.000728266354176789	0.00164745294952596
ncMCESVM	0.815600000000000	0.00638052592746950	0.002017699460056194	0.00456435328594019
PSO	0.529638941728619	0.01588637565572492	0.005023713083714171	0.01136442853616707
SVM	0.614191681390083	0.00181374011491990	0.000573554984676242	0.00129747151684398
SVM-RFE	0.863041383335182	0.00727375571032084	0.002300163518826977	0.00520333137972157

In this table we show for each classification methods (10 experiments) the mean, standard deviation, standard error of the mean and the confidence interval multiplier for standard error measures achieved.

Table 3. Cross-Validation Recall

Method	Recall	Standard deviation	Standard error	Confidence interval
FSMKL	0.99460000000000	0.000966091783079297	0.000305505046330390	0.000691100428827288
GA	0.99680000000000	0.002529822128134706	0.000800000000000001	0.001809725730238565
MKL	0.99060000000000	0.001349897115421107	0.000426874949162190	0.000965658223866368
ncMCESVM	0.91909894540534	0.004915965086298400	0.001554564657056915	0.003516669573994235
PSO	1.00000000000000	0.000000000000000000	0.000000000000000000	0.000000000000000000
SVM	0.99400000000000	0.000000000000000000	0.000000000000000000	0.000000000000000000
SVM-RFE	0.96240000000000	0.006168017869984207	0.001950498511777041	0.004412334179443671

In this table we show for each classification methods (10 experiments) the mean, standard deviation, standard error of the mean and the confidence interval multiplier for standard error measures achieved.

Table 4. Cross-Validation F-measure

Method	F-measure	Standard deviation	Standard error	Confidence interval
FSMKL	0.867134394781785	0.00179061806637700	0.000566243150919788	0.001280930999738624
GA	0.743864744638397	0.01803646723305619	0.005703631739945256	0.012902511394480350
MKL	0.785258443186657	0.00184770173029115	0.000584294590435416	0.001321766192937720
ncMCESVM	0.864251163752222	0.00490493738810264	0.001551077392692162	0.003508780833932939
PSO	0.692375234226658	0.01355243869073745	0.004285657411252063	0.009694830610163066
SVM	0.759244568203312	0.00138687018506537	0.000438566860378581	0.000992107164571326
SVM-RFE	0.909992685495729	0.00477104432601762	0.001508736688783861	0.003412999507308856

In this table we show for each classification methods (10 experiments) the mean, standard deviation, standard error of the mean and the confidence interval multiplier for standard error measures achieved.

Table 5. **Textural features selected by FSMKL during the feature selection process.**

Group	Features	Num. of Feat.
Histogram	Perc.01%, Perc.10%, Perc.50%, Perc.90% and Perc.99%	5 in 9
Absolute Gradient	GrKurtosis, GrMean, GrSkewness, GrVariance, GrNonZeros	5 in 5
Run-length Matrix		0 in 20
Co-occurrence Matrix	S(5,0)InvDfMom, S(4,0)InvDfMom, S(3,0)InvDfMom, S(0,5)InvDfMom, S(2,0)InvDfMom, S(3,3)InvDfMom, S(0,4)InvDfMom, S(4,4)InvDfMom	8 in 221
Autoregressive Model	Theta1, Theta2, Theta3, Theta4, Sigma	5 in 5
Wavelet		0 in14

Values in parenthesis represent coordinates, containing information about distance and direction between pixels. Perc. = percentile derived from the image histogram, Theta and Sigma= vector of autoregressive model, InvDfMom = inverse difference moment, Gr. = absolute gradient parameters (kurtosis, mean, skewness, variance and and percentage of pixels with nonzero gradient). FSMKL considers that Run-length matrix and wavelet textural features are not relevant for the given classification problem.

Table 6. **Inter- intra-variability in the manual spot segmentation process with ten 2D electrophoresis images.**

		Image ID									
	Iteration	1	2	3	4	5	6	7	8	9	10
Clinician_A	1	404	545	539	545	445	539	565	307	539	565
	2	433	551	527	512	412	533	579	306	533	551
Clinician_B	1	397	481	541	497	431	511	539	297	505	556
	2	401	475	512	505	429	523	545	300	471	523
Mean		408.75	513	529.75	514.75	429.25	526.20	557	302.50	512	548.75
Standard deviation		16.41	40.56	13.35	21.07	13.52	12.26	18.04	4.79	31.09	18.11

We identify each image with an Image ID number and present for each one of the two clinicians the number of spots manually segmented in two consecutive iterations. Mean and standard deviation are calculated at the bottom of the table to measure the inter- and intra-variability.

Table 7. **Textural features extracted with Mazda and used in this work.**

Group	Features	Num. of Feat.
Histogram	Mean, variance, skewness, kurtosis, percentiles 1%, 10%, 50%,90% and 99%	9
Absolute Gradient	Mean, variance, skewness, kurtosis and percentage of pixels with nonzero gradient	5
Run-length Matrix	Run-Length non-uniformity, grey-level non-uniformity, long-run emphasis, short-run emphasis and fraction of image in runs	20
Co-occurrence Matrix	Angular second moment, contrast, correlation, sum of squares, inverse difference moment, sum average, sum variance, sum entropy, entropy, difference variance and difference entropy	221
Autoregressive Model	Theta: model parameter vector, four parameters; Sigma: standard deviation of the driving noise	5
Wavelet	Energy of wavelet coefficients in sub-bands at successive scales; max four scales, each with four parameters	14

These features are based on image histogram, co-occurrence matrix (information about the grey level value distribution of pairs of pixels with a preset distance d = 1,2,3,4 and 5 pixels apart along a given direction with angle $\Theta = 0°, 45°, 90°, 135°$, run-length matrix (information about sequences of pixels with the same grey level values in a given direction), image gradients (spatial variation of grey levels values), auto-regressive models (description of texture based on statistical correlation between neighbouring pixels) and wavelet analysis (information about image frequency content at different scales).

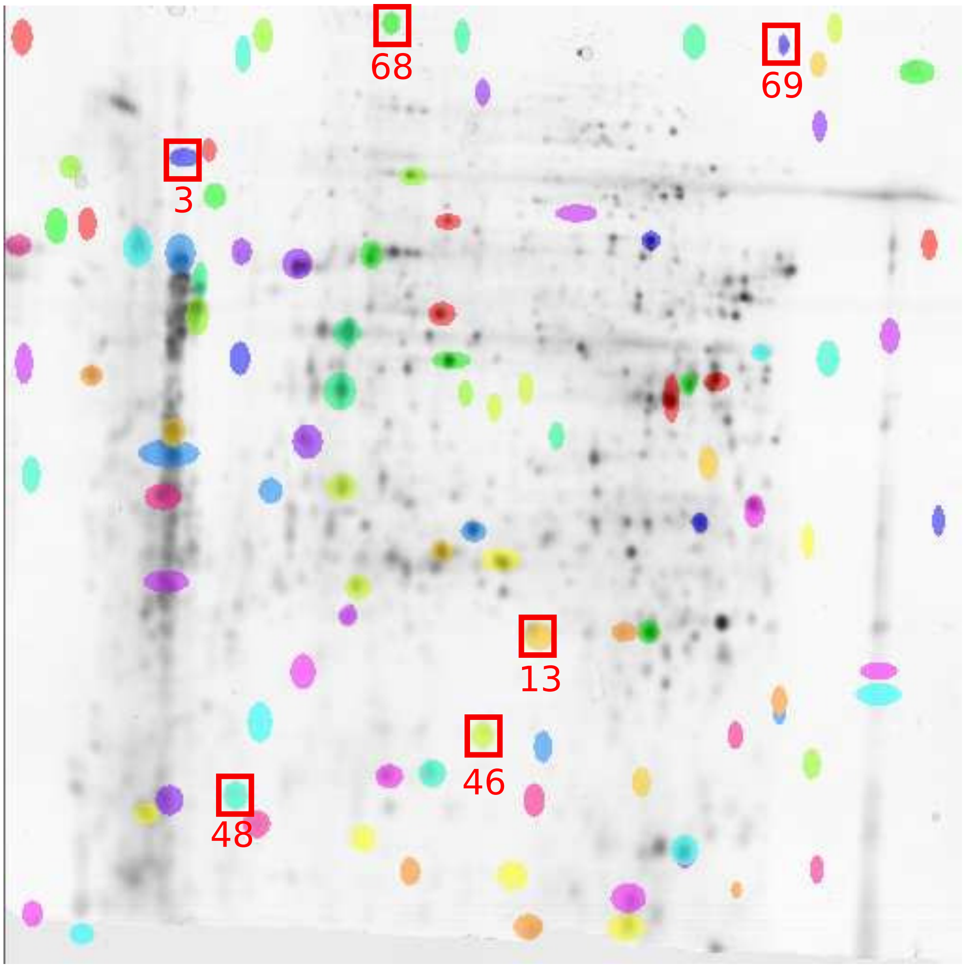

Figure 1. Best model's spots wrongly detected during the 10 experiments for the first image in the dataset. Frequency of the error for each spot is in brackets after the id: spot_3 (9), spot_13 (1), spot_46 (3), spot_48 (3), spot_68 (6), spot_69 (2).

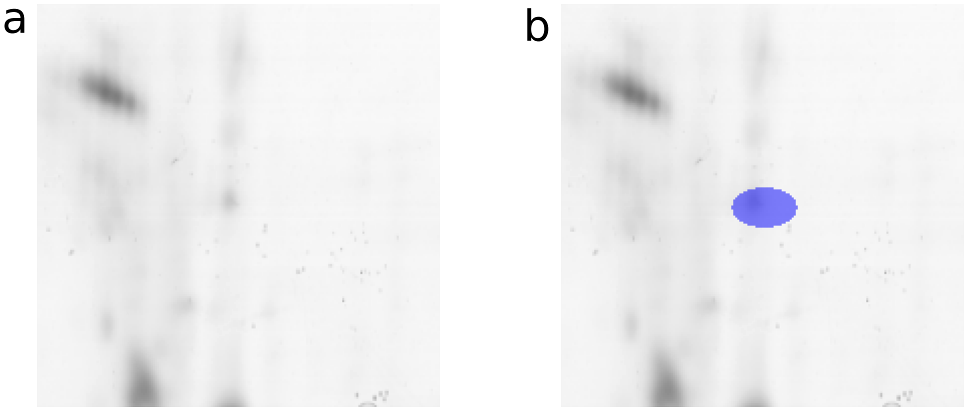

Figure 2. Spot_3-Image_1 wrongly detected in experiments: 1,2,3,4,5,6,7,8,10. False Negative: our experts marked this spot as a Protein but our technique did not found it. a) Without manual ROI b) With manual ROI

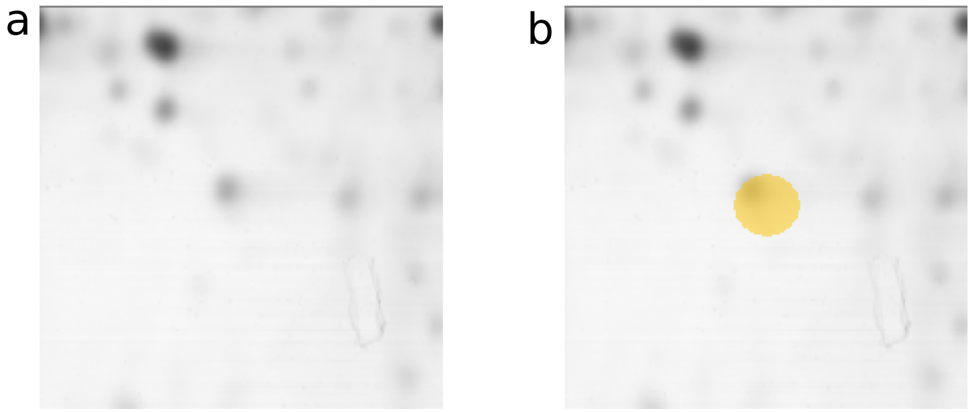

Figure 3. **Spot_13-Image_1 wrongly detected in experiments: 4. False Negative: our experts marked this spot as a Protein but our technique did not found it.** a) Without manual ROI b) With manual ROI

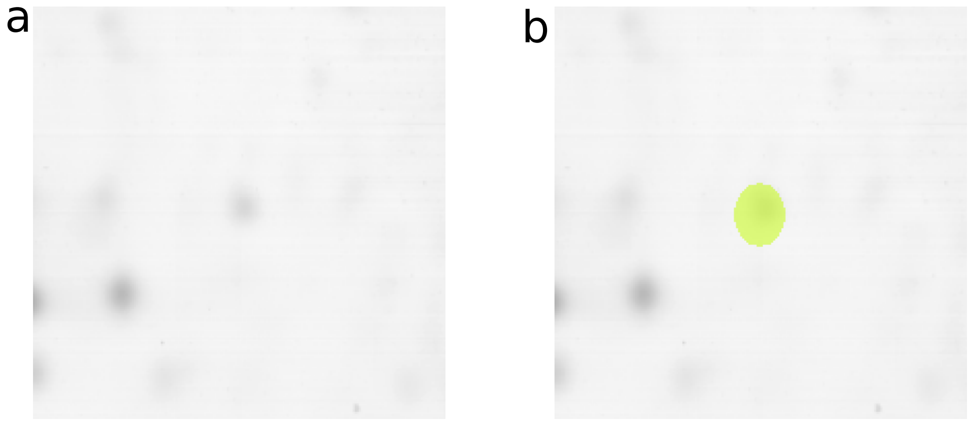

Figure 4. **Spot_46-Image_1 wrongly detected in experiments: 4,5,8. False Negative: our experts marked this spot as a Protein but our technique did not found it.** a) Without manual ROI b) With manual ROI

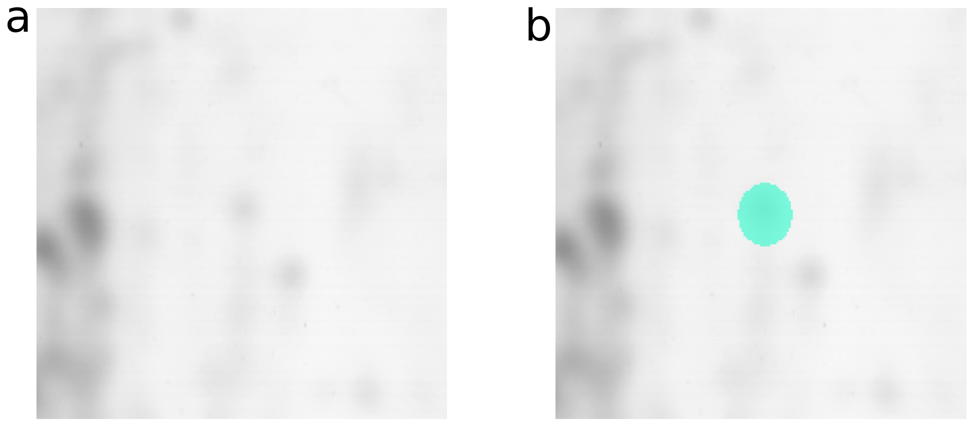

Figure 5. **Spot_48-Image_1 wrongly detected in experiments: 3,4,7. False Negative: our experts marked this spot as a Protein but our technique did not found it.** a) Without manual ROI b) With manual ROI

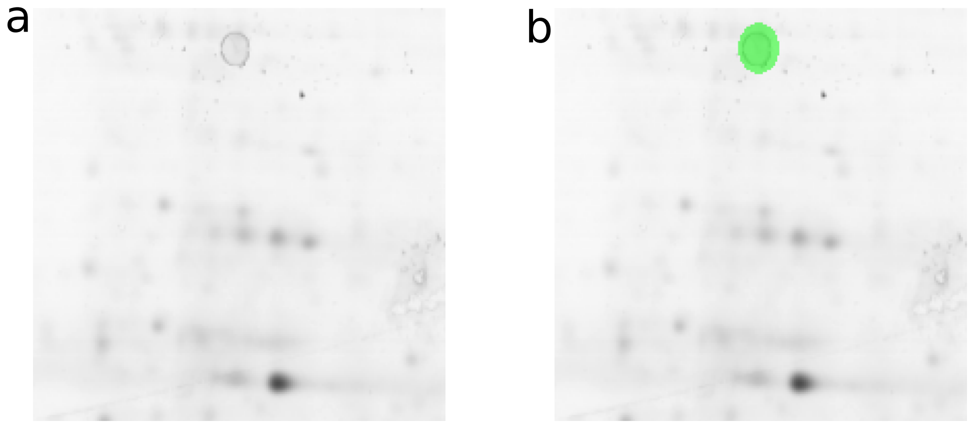

Figure 6. **Noise_68-Image_1 wrongly detected in experiments: 1,2,3,6,9,10. False Positive: our experts marked this spot as Noise but our technique did not found it.** a) Without manual ROI b) With manual ROI

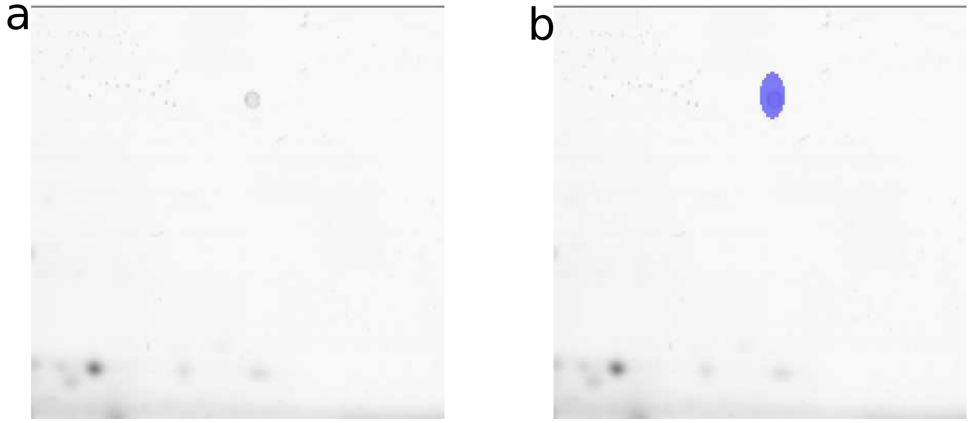

Figure 7. **Noise_69-Image_1 wrongly detected in experiments: 1,3. False Positive: our experts marked this spot as Noise but our technique did not found it.** a) Without manual ROI b) With manual ROI
